# Supplementary material for: Psychological consultation apps in Saudi Arabia: A study for experts’ evaluation and users’ points of view
Source: PLoS One. 2025 Jul 23;20(7):e0329029. doi: 10.1371/journal.pone.0329029 (PMC12286377; doi:10.1371/journal.pone.0329029)
Supplement: Attachment — Submitted filename: SMART .pdf [file pone.0329029.s001.pdf]

Dear Authors,

Thank you for your submission. The manuscript has been thoroughly reviewed, and several suggestions for improvement have been provided. Please carefully consider the feedback and revise the manuscript accordingly. Make sure to highlight all changes in the text for easier review. We look forward to receiving the revised version and appreciate your efforts to enhance the quality of your work.

Sincerely,

Abstract

This abstract provides useful information but has several issues. First, the research goal is not clearly stated, and it is not specified what exact aspects of user experience the study addresses. Additionally, the methodology is not fully explained; the concept of SMART heuristic is vague and may be unclear to readers unfamiliar with the term. Furthermore, the results are presented in a general way, without enough detail, and specific findings for each app, particularly Mind, are not clearly differentiated. The categories for positive and negative reviews are mentioned, but there is no information about the number or percentage of these reviews. Lastly, while recommendations are mentioned at the end of the abstract, they are not elaborated on, and their impact on improving the apps is not clarified.

Keywords appear below the abstract and should include five MeSH headings selected from the 'MeSH Database', available at http://www.pubmed.gov.

Introduction

The introduction of this paper provides a broad overview of the rise of mobile health technologies, including telemedicine and mental health apps, but there are several areas for improvement.

First, the introduction lacks a clear and focused thesis statement. While it mentions the importance of telemedicine, remote consultations, and the specific context of mental health, the main objective of the paper is not explicitly introduced until later. It would be helpful to more directly state the aim of evaluating psychological consultation apps in Saudi Arabia and why this research is needed.

Second, the section about the historical development of remote medicine (starting in the 1900s) feels somewhat disconnected from the focus of the paper, which is the usability of current mental health apps in Saudi Arabia. The transition from the general development of telemedicine to the specific problem of psychological consultation apps could be smoother. More emphasis should be placed on recent developments in mental health apps, particularly in Saudi Arabia, to establish a clear context for the study.

Another issue is the structure of the content. The paragraph discussing the negative impact of COVID-19 on senior citizens' mental health feels somewhat tangential to the core topic of the paper, which is evaluating app usability. While it is relevant to the broader issue of mental health, it does not directly contribute to the discussion about the need for better psychological consultation apps in Saudi Arabia.

Additionally, while the introduction mentions the Saudi government's efforts in digital health transformation (Vision 2030), the relevance of this to the research is not fully explained. How does the government's focus on digital healthcare connect to the need for usability studies of mental health apps? This could be clarified to strengthen the argument for the significance of the study.
Lastly, the literature review within the introduction is comprehensive but could benefit from better synthesis. The numerous references to previous studies on telemedicine and psychological apps seem to be a listing of studies without providing a clear comparison or synthesis of their findings. It would be more effective to highlight the gaps in the literature that this study aims to address, particularly the lack of research on psychological consultation apps in Saudi Arabia.

Method

In Study One, the authors mention that five experts were contacted for the evaluation of the apps, but the justification for selecting exactly five evaluators is not fully explained. While Nielsen's guideline suggests that 3–5 experts can find around 75% of usability issues, the rationale behind choosing this number specifically for this study, and how the evaluators were selected (e.g., their qualifications or experience), is not detailed. Providing more context on how the experts were chosen and whether their backgrounds align with the app's target audience could strengthen the methodology's credibility. While the SMART heuristics are clearly listed, the explanation of how the heuristic evaluation process was conducted is somewhat vague. For example, the text mentions a meeting with the evaluators to discuss usability issues, but it does not elaborate on the structure of these discussions or how discrepancies between evaluators were handled. More information on how consensus was reached (or not) during this discussion could clarify the reliability of the findings and reduce potential bias.
The heuristic evaluation method is a widely used and effective usability evaluation tool, but it has certain limitations. These include the subjectivity of the evaluators, the possibility of missing usability issues that real users might encounter, and the lack of interaction with actual users. While the paper briefly mentions that heuristic evaluation is "inexpensive" and "effective," it would benefit from a more balanced view, acknowledging these limitations and the potential impact they may have on the results. The second study, which uses sentiment analysis of user reviews, does not provide enough detail on the specific methodology used for text analysis. While the use of Python and LDA (Latent Dirichlet Allocation) for topic modeling is mentioned, it lacks a description of how the specific parameters for LDA were set, the number of topics generated, or how the reliability of the sentiment classification (positive vs. negative) was ensured. The steps for tokenizing and vectorizing the reviews could be further elaborated, including the handling of stopwords, stemming, or lemmatization. Without such details, it is unclear how robust the analysis is or whether it is replicable. The section mentions that user reviews were extracted from the App Store and the number of reviews (459) was "limited," but the criteria for selecting which reviews to include are not clear. For example, were reviews considered based on a specific time period, version of the app, or particular user demographics? The paper briefly touches on the filtering process based on rating scores but does not explain how the reviews were chosen, particularly

given the "early stages" of psychological apps in Saudi Arabia. A more detailed explanation of the data collection process, including how user feedback was filtered and categorized, would enhance the transparency of the research.

While it is mentioned that data cleaning was performed using Python libraries to remove redundant or missing data, no specifics are given about the techniques used (e.g., how missing data were handled or how duplicates were identified). This is an important aspect of ensuring the quality and validity of the dataset, so providing more information about this step would be beneficial. The methodology section indicates that reviews were categorized into seven categories for negative reviews and three for positive reviews, but it does not explain what these categories are or how they were derived. A more detailed description of the categories and how they were identified would clarify how the data were analyzed and whether the categories reflect meaningful insights. The process of categorization, as well as the coding process, should be explained in more detail to ensure the reliability of the findings. The paper categorizes user reviews into positive and negative based on their ratings (1–2 stars for negative, 3–5 stars for positive), but this approach may be oversimplified. It assumes that ratings directly reflect sentiment, but reviews can sometimes be more nuanced than just positive or negative. For example, a 3-star review might contain both positive and negative comments, making it more complex than a binary classification. A more sophisticated sentiment analysis approach or an additional layer of analysis could be used to provide a deeper understanding of the reviews.

Result

In Section 4.1, the results of the heuristic evaluation are presented in Table 2. However, the analysis of the results does not include any statistical tests or comparison of the severity ratings between apps. The study simply presents the total and average severity ratings for each app, but there is no indication of whether these differences are statistically significant. For instance, the Mind app has the highest severity rating (70), and Labyh the lowest (44), but the study does not explain whether this difference is meaningful or if it could be due to random variation. The lack of statistical analysis undermines the strength of the conclusions drawn from these ratings. Including measures of variance, confidence intervals, or even basic descriptive statistics would help assess the reliability and significance of these results.

The discussion of the severity ratings for each heuristic in Section 4.1 is somewhat unclear and inconsistent. For example, the paper states that "SMART 5 and SMART 10 received the highest ratings across all apps," but it is unclear whether this means these heuristics were rated poorly (high ratings indicating severe issues) or well. A more detailed interpretation of what "high" ratings mean in the context of each heuristic would be useful. Furthermore, the narrative about which heuristics were problematic for which apps is fragmented and could be better organized. For example, it states that the Mind app received high ratings in areas like "providing notifications" and "poor error prevention," but a clearer explanation of how these findings relate to specific usability problems would enhance the reader’s understanding of the usability issues identified.

In Section 4.2, the categorization of user reviews into "positive" and "negative" categories is too simplistic and does not capture the complexity of user feedback. The paper uses a binary classification of reviews (positive vs. negative), but sentiment in user reviews is often more nuanced. For example, a review might contain mixed sentiments—such as praise for certain features but criticism for others. By categorizing reviews solely into two groups based on their star ratings (1–2 for negative and 3–5 for positive), the analysis loses valuable detail. A more advanced sentiment analysis method or multi-dimensional categorization would allow for a deeper understanding of user opinions.

Additionally, while the authors present various categories for negative reviews (e.g., "general dissatisfaction," "booking and payment issues," "access issues," etc.), the explanations of these categories are somewhat vague. Each category is introduced with broad descriptions, but specific examples of how user feedback was classified into these categories would help make the process more transparent and replicable. Moreover, the paper does not discuss how these categories were developed or how consistent the classification process was among reviewers. Providing more details on the coding process and inter-rater reliability would improve the robustness of the results.

The paper states that "the number of negatives was seven, while the number of positives was three," but this is presented without context. Are these counts based on a specific time period or app version? What is the total number of reviews analyzed for each app, and how were these seven negative and three positive reviews selected? The methodology section mentions that the total number of reviews for all three apps was 459, but there is no explanation of how many reviews were considered for each individual app and how the selection was made. A clearer explanation of how these reviews were chosen, and why some were categorized as positive and others negative, would be helpful. Additionally, the small number of positive reviews (three) in comparison to the negative ones (seven) seems unusual, and more context is needed to understand this disparity—whether it is reflective of the overall user sentiment or a result of sampling bias.

The review categories for both positive and negative reviews appear somewhat redundant. For example, the "general dissatisfaction" category includes feedback about the app's overall quality, while other categories like "access issues," "interface concerns," and "functionality issues" can also overlap with general dissatisfaction. A more hierarchical categorization could avoid redundancy and make the findings clearer. For instance, a broad "app performance" category could subsume the "access issues," "interface concerns," and "functionality issues," while also providing sub-categories for specific problems under each aspect.

Throughout the results, there is inconsistent or unclear terminology that may confuse readers. For example, when discussing the categories of user reviews, terms like "general dissatisfaction" and "feature limitations" are used without explaining what exactly qualifies as "general dissatisfaction" or how this term is operationalized. Similarly, the term "amazing app idea" in positive reviews is somewhat vague and lacks specific user feedback to justify its inclusion in this category. Providing clear definitions for these categories would help ensure that the reader understands what is meant by each term and how it was applied in the analysis. The presentation of results in Figure 2 (regarding the user reviews) could be enhanced by providing more quantitative data or visual

representations. While categories are listed, there are no charts, graphs, or other visual aids to help readers easily understand the distribution of positive and negative reviews across different categories. Simple bar charts, pie charts, or even heatmaps could help make the review analysis more digestible and provide a more intuitive grasp of the findings.

Discussion

One of the main issues in the Discussion section is the lack of deep analysis and interpretation of the results. While the negative and positive aspects are highlighted, there is limited explanation of why these issues exist or what specific factors contributed to them. For instance, the Mind app is mentioned as needing improvement, receiving a severity rating of 70, but no detailed analysis is provided on what led to these problems or how they can be rectified. A deeper analysis of the causes behind these usability issues could help developers understand the root of the problems and devise more effective solutions. The Discussion section does not address the limitations of the study. Usability studies typically have limitations, such as sample size, time constraints, or user-specific factors. Acknowledging these limitations would help readers evaluate the results more realistically. Additionally, the study appears to be conducted in a specific region (Saudi Arabia), and this could influence the applicability of the results in other regions or contexts. Mentioning this limitation could have provided more context for understanding the scope and relevance of the findings.

Although the discussion briefly mentions usability problems like "focusing on one task" and "error prevention," there is no detailed exploration of how these issues affect the user experience and what specific solutions might address them. For example, why users find the interface of the Mind app difficult to navigate is not explored in depth, nor is there a discussion of how this could be remedied. Providing concrete examples from the results would have helped clarify how these issues manifest in real usage and what practical solutions might be effective. The discussion briefly compares the three apps (Labyh, Estenarah, and Mind), but the comparison is quite superficial and lacks in-depth analysis. For example, while the Mind app has been identified as having more usability issues, the discussion does not explore which specific features or functions contribute to these problems. A more detailed comparison of the apps based on the results would have helped pinpoint the strengths and weaknesses of each app, providing more actionable insights for designers.

In the Discussion section, general concepts like "responsive design" or "error prevention" are mentioned, but there is little reference to existing research or studies that support these concepts. While references were made in the Methodology section, the Discussion section would benefit from citing studies that reinforce the importance of the highlighted issues. This would have lent credibility to the suggestions and helped frame the findings within the context of broader usability research. The recommendations provided in the section are generally useful but remain quite broad and unspecific. For instance, recommending "simple user interfaces" or "error prevention" is helpful, but it does not offer practical, detailed solutions or examples for how to implement these strategies. More specific suggestions, such as how to streamline user flows or improve error

message design, would have been more beneficial. Offering step-by-step guidelines or best practices could help designers understand how to improve the apps more effectively. While the importance of "personalization" and "adaptive interfaces" is noted, there is no detailed examination of the diverse needs of users, particularly from cultural or demographic perspectives. In a country like Saudi Arabia, where there is considerable cultural and social diversity, user needs may vary significantly. The study does not address these differences, which could have influenced the usability challenges. A more detailed analysis of how user needs differ across cultural or demographic groups could have provided more targeted recommendations for improving the apps.
