# Supplementary material for: Psychological consultation apps in Saudi Arabia: A study for experts’ evaluation and users’ points of view
Source: PLoS One. 2025 Jul 23;20(7):e0329029. doi: 10.1371/journal.pone.0329029 (PMC12286377; doi:10.1371/journal.pone.0329029)
Supplement: Attachment — Submitted filename: Response to Reviwers.pdf [file pone.0329029.s003.pdf]

**Reviewer #1 Comment A.1**: Introduction Section. Tighten the narrative flow by placing a clear, concise statement of the research gap earlier in the introduction — ideally before the research questions and objectives are introduced.

**AuthorResponse:** Thanks for this observation. We have revised the introduction to improve narrative flow by positioning the research gap after the literature review and immediately before the study aim and objectives (p. 3, paragraph 3). This placement allows the gap to emerge logically from prior work, ensuring that it is both well-supported and clearly framed. The paragraph beginning with "However, despite these contributions, research on psychological consultation apps in Saudi Arabia remains sparse" now summarizes the key limitations in the literature and transitions directly into our study's contribution.

**Reviewer #1 Comment A.2:** Introduction Section. Clearly state what is missing in the current literature. For example:

• Have previous studies not focused on mental health apps in the Saudi Arabian context?
• Is there a lack of comparative usability analysis using real user reviews?
• Are cultural and contextual usability factors underexplored in prior research?

**Author Response:** We have explicitly addressed this concern in paragraph 3 of the revised introduction (p. 3). The revised text clearly identifies that:

- Few studies focus specifically on psychological consultation apps.
- There is limited use of real user feedback to assess usability.
- The cultural and linguistic needs of Saudi users are underexplored.
- There is a lack of comparative analysis that integrates heuristic evaluation with sentiment analysis of actual users. These points are presented together to sharpen the articulation of the research gap.

**Reviewer #1 Comment A.3:** Introduction Section. Follow the gap with a direct explanation of why this study matters: Emphasize how addressing this gap will contribute new knowledge, practical value, or methodological contribution.

**AuthorResponse:** Thanks for this comment. We have addressed this in paragraph 4 of the revised introduction (p. 3). The study's relevance is now emphasized by stating that it evaluates three apps (Labayh, Mind, and Estenarah) using a dual-method approach that integrates heuristic evaluation and sentiment analysis. This contributes methodologically by combining expert and user perspectives, and practically by producing culturally grounded recommendations for app designers and healthcare policymakers in Saudi Arabia.

**Reviewer #1 Comment B.1:** Discussion Section 1) Structural Organization. The discussion section would benefit from a clearer structural organization. Currently, it merges results, analysis, and recommendations in a way that can obscure the key insights of the study. I recommend dividing this section into clear thematic sub-sections, such as:

• Summary of Key Findings
• Usability Challenges (with potential sub-themes like error prevention, interface design, etc.)
• Positive Aspects
• Recommendations for Improvement
• Implications for Mental Health App Design in Saudi Arabia

**Author Response:** We appreciate this suggestion. We restructured the entire Discussion section (Section 5) into the exact five sub-sections recommended by the reviewer: 5.1 Summary of Key

Findings, 5.2 Usability Challenges, 5.3 Positive Aspects, 5.4 Evidence-Based Recommendations, and 5.5 Implications for Mental Health App Design in Saudi Arabia. This reorganization enhances clarity and ensures a more coherent presentation of the study's insights.

**Reviewer #1 Comment B.2:** Discussion Section 2) Integration with Literature. While the discussion references relevant literature, there is often a lack of clear integration between the empirical findings and these theoretical insights. I suggest more explicitly linking findings from the current usability evaluations with the literature cited. Example Revision: Instead of stating: 'Research indicates that effective error messages are crucial for user satisfaction...'. It would be stronger to write: "Our findings indicate that Mind's ambiguous error messages negatively affected user experience. This aligns with Smith et al. [74], who argue that clearly phrased, contextual error feedback enhances user satisfaction and system transparency."
**Author Response:** We agree with the reviewer that stronger integration was needed. In the revised Discussion section (particularly 5.2 Usability Challenges and 5.3 Positive Aspects), we enhanced the alignment between empirical findings and relevant literature. For example, we explicitly link Mind's ambiguous error messaging to prior findings by Nielsen [74], and we expanded this integration to include a wide range of supporting studies. To strengthen this section further, we have now incorporated references [74–96], covering topics such as cognitive load [77], mobile usability heuristics [76], adaptive UI design [78], engagement aesthetics [75, 87, 88], testing practices [82–84], backend optimization [85, 86], and cultural usability in Saudi Arabia [96]. This enriched literature support ensures that the discussion is analytically grounded and theoretically informed throughout.

**Reviewer #1 Comment B.3:** Discussion Section 3) Comparative Analysis of the Three Apps. The comparative analysis between the three apps—Labyh, Mind, and Estenarah—could be further developed. While comparative statements exist, they often remain descriptive rather than analytical. Expand on why one app succeeded or failed relative to the others..."
**Author Response:** Thank you for this insightful comment. We enhanced the comparative depth throughout Sections 5.2 and 5.4 by discussing the design choices and implementation strategies that contributed to differences in usability. For example, we analyzed how Labyh's cleaner navigation contributed to better task performance, while Estenarah's simplicity came at the cost of feature limitations. These analyses help explain not only what happened but why usability scores diverged between apps.

**Reviewer #1 Comment B.4:** Discussion Section 4) Repetition of Themes. There is noticeable repetition of some themes, such as task overload, error prevention, and interface challenges, across multiple sections. Consolidating overlapping discussions could enhance clarity and allow more space for nuanced interpretation.
**Author Response:** We carefully reviewed and revised the entire Discussion section to eliminate repetition. Related topics were consolidated under broader labels within 5.2 Usability Challenges (e.g., error messaging and terminology are now combined under "Error Prevention and Messaging"). This refinement reduces redundancy and enhances thematic clarity.

**Reviewer #1 Comment B.5:** Discussion Section 5) Descriptive vs Evaluative Tone. Some parts of the discussion adopt a descriptive tone, which weakens the analytical rigor of the section. Suggestion: Adopt a more evaluative tone to critically reflect on the findings.

**Author Response:** We revised multiple passages across the Discussion to adopt a more evaluative and critical tone. For instance, we now explicitly state how Labyh's failure to adapt across devices reveals a significant design flaw despite otherwise strong performance. The revised text presents more analytical reasoning, in line with the reviewer's example.

**Reviewer#1 Comment#C:** (Recommendation Section). The recommendations section provides a series of general usability best practices — such as improving error messaging, implementing responsive design, and supporting diverse payment options — which are certainly relevant to the design of psychological consultation apps. However, as it stands, this section lacks a clear, direct link to the empirical findings presented in the earlier parts of the paper. While the study includes valuable data on usability issues derived from user reviews and comparative app analysis, these insights are not explicitly reflected in the recommendations. This results in a disconnect between the evidence gathered and the guidance offered. For instance, suggestions such as "conduct user journey mapping" or "implement contextual adaptation" are useful but appear to be generic UX advice rather than recommendations tailored to the actual usability problems uncovered in Mind, Labyh, and Estenarah. Suggestion for Improvement:
To enhance the value and academic rigor of the recommendations, I strongly encourage the authors to restructure this section to reflect the comparative analysis presented earlier. One effective approach would be to build a matrix that clearly maps:
• Usability problems observed in each app
• Comparative insights (e.g., where one app succeeded and another failed)
• Targeted, app-specific recommendations grounded in these observations
**Author Response:** We appreciate this suggestion and have removed the standalone recommendations section. Instead, we integrated a detailed, evidence-based and app-specific set of recommendations directly into the Discussion section under Subsection 5.4 – Evidence-Based Recommendations. This subsection:

- Links each recommendation explicitly to the findings of the heuristic evaluation and user sentiment analysis.
- Identifies which app(s) the issue pertains to.
- Follows a structured format resembling the suggested matrix (Issue → App → Recommendation).

**Reviewer#1 Comment#D:** (Conclusion Section). While the conclusion effectively summarizes the key findings, it tends to be overly descriptive and lacks synthesis. Rather than reiterating the results, the conclusion should offer a deeper interpretation of their broader significance — essentially answering the "so what?" of the study. Additionally, the paragraph addresses multiple themes (usability findings, future directions, methodological limitations, sentiment analysis critiques, and reviewer demographics) within a single block, which dilutes clarity and impact. Consider separating the conclusion into clearer thematic segments. Start with a concise summary of the main findings and their implications, followed by a discussion of methodological limitations, and conclude with forward-looking insights or practical recommendations. The unique contribution of the study — such as the integration of heuristic evaluation and sentiment analysis within a culturally specific context (Saudi Arabia) — should be highlighted more explicitly.
**Author Response:** We appreciate the reviewer's insightful feedback. In response, we revised the conclusion paragraph to move beyond description and offer a more analytical synthesis of the findings. The revised paragraph now emphasizes the broader significance of the study, clearly

articulates its unique methodological contribution, namely (the integration of heuristic evaluation and sentiment analysis in a culturally specific context), and includes a concise reflection on limitations and directions for future work. While we maintained a unified paragraph format for flow, we ensured the paragraph addresses each theme explicitly.

**Reviewer #3 Comment#1:** Are you sure the sentiment analysis falls under the category of quantitative approach? The categorization to into several sub-themes such as general dissatisfaction, booking and payment issues, access issues, functionality errors, interface concerns, app performance, and feature limitations (for negative reviews) and general appreciation, praise for the app idea, and recommendations for improvement (for positive reviews) based on the subjective reviews strongly indicates qualitative analysis. How are the percentages of each category as shown in Fig 2 and 3 obtained?
**Author Response:** Thank you for this valuable observation. We acknowledge that the sub-themes identified are interpretable in a qualitative sense; however, we clarify that the sentiment analysis methodology applied in this study is quantitative in nature. Specifically, we used CountVectorizer to convert the preprocessed review texts into a numerical document-term matrix. This matrix was then processed using Latent Dirichlet Allocation (LDA), a probabilistic topic modeling technique that generated topic distributions across the corpus. Each review was automatically assigned to its most probable topic based on posterior probabilities generated by the LDA model. The percentages shown in Figures 2 and 3 reflect the proportional distribution of reviews assigned to each topic. This explanation has now been added to the end of Section 3.2 to clarify the quantitative nature of the analysis and the method used to calculate topic-wise distributions.
