# Supplementary material for: Psychological consultation apps in Saudi Arabia: A study for experts’ evaluation and users’ points of view
Source: PLoS One. 2025 Jul 23;20(7):e0329029. doi: 10.1371/journal.pone.0329029 (PMC12286377; doi:10.1371/journal.pone.0329029)
Supplement: Attachment — Submitted filename: Response to Reviewers.pdf [file pone.0329029.s004.pdf]

Reviewer #1 Comment 1: While the structure of the revisions could be improved, the authors have addressed all reviewer comments satisfactorily, and the responses are acceptable.
Author Response: We thank the reviewer for acknowledging that all comments have been satisfactorily addressed. Regarding the observation about the "structure of the revisions," we believe this may refer to the consistency and clarity of the revised text following the incorporation of all reviewer suggestions. To strengthen the final presentation, we engaged a professional proofreading service (PaperTrue), which made over 780 language and structure-related improvements to the manuscript (380 insertions, 384 deletions) across a 6,498-word document. These changes addressed grammar, cohesion, sentence structure, and consistency, while ensuring that the content remained faithful to the revised structure. We hope these enhancements improve the overall clarity and readability of the manuscript and address any remaining structural concerns.

Editor Comment: Following a comprehensive review, your paper has been acknowledged for its substantial contribution to the field of health sciences. Your research offers valuable insights into the usability of psychological consultation apps, and we are confident that it will have a significant impact for designers of psychological consultation apps, as well as practical applications aimed at improving the user experience of psychological consultation apps in Saudi Arabia
Author Response: We sincerely thank you for your kind feedback and for acknowledging the contribution of our work. We are grateful for the opportunity to share our research through your journal and are encouraged by your recognition of its value to the field of health sciences. We hope that our findings will indeed support both researchers and practitioners seeking to improve the usability of psychological consultation apps, particularly within the Saudi Arabian context. We appreciate the editorial guidance and constructive reviewer feedback that helped us refine and strengthen the manuscript.
