# Supplementary material for: Psychological consultation apps in Saudi Arabia: A study for experts’ evaluation and users’ points of view
Source: PLoS One. 2025 Jul 23;20(7):e0329029. doi: 10.1371/journal.pone.0329029 (PMC12286377; doi:10.1371/journal.pone.0329029)
Supplement: Attachment — Submitted filename: Response to Reviewers.docx [file pone.0329029.s002.docx]

Dear Reviewers,

Thank you for your thorough review of our manuscript titled "Psychological Consultation Apps in Saudi Arabia: A Study for Experts’ Evaluation and Users’ Points of View" (Manuscript Number: PONE-D-24-40629). We appreciate your valuable feedback, which has helped us improve the quality of our work. Below, we address each of your comments and outline the changes made in the revised manuscript.

**Reviewer #1:**

**Comment 1:** The introduction is too broad and lacks a clear direction toward the research problem.

**Response:** Thanks for this comment. We moved two paragraphs to a new section (1.1) and removed two broad paragraphs. We also advanced the discussion of the research objectives to an earlier point in the text.

**Comment 2:** The research gap is not explicitly described, making it difficult to understand the study's contribution.

**Response:** Thanks for this comment. We completely wrote the part that describes the research gap again. We removed broad references and added references belonging to mental health and Saudi Arabia. We discussed the research gap in a way that makes it specific and clear. We also added one paragraph about the research contribution.

**Comment 3:** Figures 1 and 2 are mentioned but not included in the paper, which disrupts the flow and leaves readers without critical visual information.

**Response:** They were included. The other reviewer saw them and commented on their clarity. We redesigned Figure 1 and included more information. We redesigned Figure 2 and divided it into Figure 2 (negative reviews) and Figure 3 (positive reviews). We added a percentage for every review category.

**Comment 4:** The process of generating the results is insufficiently detailed, leaving gaps that confuse readers and hinder their understanding of the methodology.

**Response:** Thank you for your comment. We have rewritten the methodology section to include more detailed explanations and have enhanced Figure 1 for improved visual clarity. We believe these revisions will address the gaps you identified and enhance the overall understanding of our methodology.

**Comment 5:** Subsection 4.2 does not adequately represent sentiment analysis. It appears to focus on qualitative categorization rather than the expected complex sentiment analysis process, which needs clarification.

**Response:** Thank you for your valuable feedback. We have revised this section to provide a clearer representation of the sentiment analysis process. While we acknowledge the qualitative categorization of reviews, we have emphasized the underlying complex methodologies used in our analysis. The revisions now include a more detailed explanation of our preprocessing techniques, including tokenization and vectorization, which convert text data into a numerical format suitable for analysis. Additionally, we have highlighted the use of the Latent Dirichlet Allocation (LDA) model to identify topics based on word distributions, which adds depth to our sentiment analysis approach. We believe these clarifications will provide a more comprehensive understanding of the sentiment analysis conducted and address the complexities involved.

**Comment 6:** The transition to the discussion section is abrupt and confusing. The link between the previous sections and the discussion is weak, and the structure requires significant improvement for better coherence.

**Response:** Thank you for your valuable feedback. We have added a new introductory paragraph for the discussion section that summarizes the findings from both studies. This paragraph highlights the usability evaluations of the apps and incorporates user feedback, creating a comprehensive overview. Additionally, we have made improvements to the methodology and results sections to ensure clarity and coherence. These enhancements facilitate a smoother and more logical transition into the discussion, allowing readers to better understand the implications of the findings. We believe these changes significantly strengthen the overall flow of the paper.

**Comment 7:** While the recommendations are insightful, they should align more closely with the results to provide a stronger foundation for their validity.

**Response:** We have revised the recommendations section to ensure a clearer connection between our findings and proposed solutions. Each recommendation now explicitly references specific results from our evaluations, providing a stronger foundation for their validity.
 **Comment 8:** The conclusion is overly simplistic and does not sufficiently elaborate on the findings. It should answer the research questions more explicitly and synthesize the results in greater detail.

**Response:** Thank you for your insightful comment. We acknowledge that the previous version was overly simplistic and did not delve deeply enough into the findings. In the revised conclusion, we have made a concerted effort to explicitly answer the research questions, synthesizing the results in detail. We highlighted the usability challenges faced by the apps and provided actionable recommendations based on our findings. We incorporated future directions that suggest areas for further research. This enriched conclusion aims to better encapsulate the essence of our research and its implications for the design and functionality of psychological consultation apps in Saudi Arabia.

**Reviewer #2:**

**Abstract**

**Comment 1:** The research goal is not clearly stated, and it is not specified what exact aspects of user experience the study addresses.

**Response:** Thank you for your feedback. We have clarified the research goal in the revised abstract. The aim is now explicitly stated as evaluating the user experience of psychological consultation apps in Saudi Arabia, focusing on usability aspects and user satisfaction. This should provide a clearer understanding of the study's objectives.

**Comment 2:** The methodology is not fully explained; the concept of SMART heuristic is vague.

**Response:** We appreciate your comment. In the improved abstract, we have elaborated on the SMART heuristic framework, explaining its purpose in assessing usability by identifying issues based on established principles.

**Comment 3:** The results are presented in a general way, without enough detail.

**Response:** Thank you for this comment. We have enhanced the presentation of results in the revised abstract, providing specific findings, such as the severity rating of the "Mind" app and the key usability issues identified.

**Comment 4:** Recommendations are not elaborated on, and their impact on improving the apps is not clarified.

**Response:** We acknowledge your concern. In the updated abstract, we have elaborated on the recommendations for improvement, specifying actions such as enhancing task-focused design and increasing adaptability for diverse mobile environments. This addition aims to clarify their potential impact on improving the apps.

**Introduction**

**Comment 5:** The introduction lacks a clear and focused thesis statement. The main objective

of the paper is not explicitly introduced until later

**Response:** Thanks for this comment. We went through the introduction again and narrowed it by moving two paragraphs to section (1.1) and removing two very broad paragraphs. We also advanced the discussion of the research objectives to an earlier point in the text. These modifications should make reaching the main objective fast and smooth.

**Comment 6:** The section about the historical development of remote medicine feels disconnected.

**Response:** Thanks for this comment. We moved this paragraph to section (2.1).

**Comment 7:** The paragraph discussing the negative impact of COVID-19 on senior citizens' mental health feels somewhat tangential to the core topic of the paper, which is evaluating app usability. While it is relevant to the broader issue of mental health, it does not directly contribute to the discussion about the need for better psychological consultation apps in Saudi Arabia.

**Response:** Thank you for this comment. Yes, we read it again and find it very broad and does not directly contribute to the discussion. Therefore, we removed this paragraph from the manuscript.

**Comment 8:** While the introduction mentions the Saudi government’s efforts in digital health

transformation (Vision 2030), the relevance of this to the research is not fully explained. How does the government's focus on digital healthcare connect to the need for usability studies of mental health apps? This could be clarified to strengthen the argument for the significance of the study.

**Response:** Thanks for this comment. We fixed this issue by strengthening the argument for the connection of this part with the aim of our study. We removed the part that talks about Seha since it lacks a connection to our study goal.

**Comment 9:** The numerous references to previous studies on telemedicine and psychological apps seem to be a listing of studies without providing a clear comparison or synthesis of their findings. It would be more effective to highlight the gaps in the literature that this study aims to address, particularly the lack of research on psychological consultation apps in Saudi Arabia.

**Response:** Thanks for this comment. We revised this part. We removed broad references and added focused ones that are related to mental health and Saudi Arabia. We also compared the findings of these studies. We wrote this part again in a way that made the research gap very specific and clear.

**Method**

**Comment 10:** The justification for contacting exactly five experts for app evaluation is insufficiently explained.
**Response:** We added a justification for the selection of five evaluators, referencing Nielsen's guidelines and detailing the evaluators’ qualifications.

**Comment 11:** More context on experts’ qualifications and selection criteria is needed. **Response:** Thank you for your comment. We have added details about the experts' qualifications and the selection criteria.

**Comment 12:** The heuristic evaluation process explanation is vague; details on evaluator discussions are lacking.
**Response:** Thank you for your comment. We have clarified the heuristic evaluation process, including details about the individual meetings held with evaluators to discuss and clarify identified usability issues.

**Comment 13:** Acknowledge the limitations of the heuristic evaluation method regarding subjectivity and real user interaction.
**Response:** Thank you for your comment. We have acknowledged the limitations of the heuristic evaluation method to provide a balanced view.

**Comment 14:** Insufficient detail on sentiment analysis methodology; parameters for LDA not described.
**Response:** Thank you for your comment. We have added details about the sentiment analysis methodology, including the key parameters for LDA and the validation process.

**Comment 15:** The steps for tokenizing and vectorizing the reviews could be further elaborated, including the handling of stopwords, stemming, or lemmatization.
**Response:** We have elaborated on the steps for tokenizing and vectorizing the reviews, including the use of Count Vectorizer, LDA parameters, and the process for identifying key topics and validating the sentiment classification.

**Comment 16:** Data collection criteria for user reviews are unclear; specifics on the filtering process and review selection are needed.
**Response:** Thank you for your comment. By "limited," we mean that the number of user reviews was small, and all available reviews were included in the analysis without any filtering or categorization.

**Comment 17:** Details on data cleaning methods using Python libraries not provided. **Response:** We have provided additional details on the techniques used, including the library used, handling missing data, removing duplicates, categorizing reviews, and validating the dataset.

**Comment 18:** Categories for reviews (seven negative, three positive) need clearer explanation and derivation.
**Response:** Thank you for your feedback. We have updated the methodology section to include a detailed explanation of how the categories were derived.

**Comment 19:** The binary classification of reviews oversimplifies sentiment; a more nuanced approach is suggested.
**Response:** Thank you for your feedback. Our approach aligns with established practices in exploratory studies where rating-based classification provides a straightforward method to identify general sentiment trends. While we acknowledge the nuances in some reviews, this method is practical for capturing broad patterns. Future work can incorporate advanced sentiment analysis for deeper insights.

**Results**

**Comment 20:** Heuristic evaluation results lack statistical analysis; no significance tests were conducted.
**Response:** The primary aim of our study was to explore usability issues and trends rather than conduct a statistical comparison. The descriptive analysis provided sufficient insights to identify the apps with the most significant usability concerns and allowed us to offer targeted recommendations for improvement.

**Comment 21:** Interpretation of severity ratings needs clarity; what "high" ratings mean should be explained.
**Response:** Thank you for your comment. We provided a detailed interpretation of what "high" ratings mean.

**Comment 22:** Fragmented narrative on heuristics and their usability issues; needs better organization. **Response:** Thank you for your feedback. We have reorganized Section 4.1 by grouping findings by app and providing detailed explanations of specific heuristics.

**Comment 23:** Vague explanations of negative review categories; specific examples are needed for clarity.
**Response:** Thank you for your comment. The paper already includes descriptions and examples for each category. However, we have revised the text to make the examples more significant and added further clarification about the classification.

**Comment 24:** Counts of negative and positive reviews lack context; methodology for selection unclear.
**Response:** Thank you for your feedback. We have revised the methodology section to include the number of reviews for each app. Additionally, we clarified that we included all available reviews up to September 2023 without applying a specific timeframe.

**Comment 25**: Redundancy in review categories; hierarchical categorization could improve clarity.
**Response:** Thank you for your suggestion. We acknowledge the potential for overlap in our categories; however, our approach aimed to maintain specificity by highlighting specific themes such as "access issues" and "interface concerns" separately. This allows us to provide a more detailed and actionable analysis of user feedback. Reorganizing the categories into a hierarchical structure at this stage could affect the consistency of our findings and recommendations. However, we appreciate your insight and will consider exploring this approach in future studies to improve clarity.

**Comment 26:** Inconsistent terminology used; definitions for categories needed for better understanding.
**Response:** Thank you for your comment. We have revised the results section to include clearer definitions for each category of user reviews. We also provided specific examples of user feedback.

**Comment 27:** The presentation of results could benefit from visual representations (charts/graphs) for clarity.
**Response:** Thank you for your comment. We have updated the representation of the results by creating a pie chart that displays the percentage distribution for each category.

**Discussion**

**Comment 26**: The lack of deep analysis and interpretation of the results.

**Response**: The revised discussion now includes a more thorough analysis of the usability challenges faced by the "Mind" app, detailing specific issues such as unclear navigation and poor error prevention. The explanation of how these factors contribute to user frustration was expanded.

**Comment 27**: No detailed analysis is provided on what led to these problems or how they can be rectified.

**Response**: The revised text now includes specific factors contributing to the usability issues in the "Mind" app, such as vague error messages and the need for a task-focused design. Recommendations for improvement were also incorporated.

**Comment 28**: The discussion does not address the limitations of the study.

**Response**: A section addressing the limitations of the study was added, acknowledging the sample size and potential biases in user reviews, as well as the limited generalizability of the findings.

**Comment 29**: The discussion briefly mentions usability problems but lacks exploration of how these issues affect user experience.

**Response**: The revised discussion now details how specific usability challenges, such as error prevention and navigation clarity, affect overall user experience, including user frustration and potential abandonment of the app.

**Comment 30**: The comparison of the three apps is superficial and lacks in-depth analysis.

**Response**: The revised discussion includes a more detailed comparison of the strengths and weaknesses of each app, with specific features and functions highlighted to clarify the differences.

**Comment 31**: General concepts like 'responsive design' or 'error prevention' are mentioned, but there is little reference to existing research.

**Response**: References to existing research supporting the importance of effective error messaging and responsive design were included to lend credibility to the findings and suggestions.

**Comment 32:** The recommendations provided in the section are generally useful but remain quite broad and unspecific. For instance, recommending 'simple user interfaces' or 'error prevention' is helpful, but it does not offer practical, detailed solutions or examples for how to implement these strategies. More specific suggestions, such as how to streamline user flows or improve error message design, would have been more beneficial. Offering step-by-step guidelines or best practices could help designers understand how to improve the apps more effectively. While the importance of 'personalization' and 'adaptive interfaces' is noted, there is no detailed examination of the diverse needs of users, particularly from cultural or demographic perspectives. In a country like Saudi Arabia, where there is considerable cultural and social diversity, user needs may vary significantly. The study does not address these differences, which could have influenced the usability challenges.

**Response:** Thank you for your thoughtful and constructive feedback regarding the recommendations section. We appreciate your insights, which emphasize the need for specificity and consideration of cultural diversity in our suggestions. In response to your comments, we have revised the recommendations to include more detailed, practical solutions, and examples for implementation.

**Reviewer #3:** 

**Comment 1:** Line 25, what does m-health stand for?

**Response:** Thanks for this comment. We changed it to mobile health.

**Comment 2:** Methodology. Does this study fall into the category of qualitative or quantitative research?

**Response:** Thanks for this comment. We added an introductory paragraph in the methodology section to explain this. This research employs a mixed-methods approach, incorporating both qualitative and quantitative elements. The heuristic evaluation represents a qualitative assessment, focusing on expert evaluations of usability and user experience based on established heuristics. In contrast, the sentiment analysis of user reviews provides quantitative data. The sentiment analysis in this study is classified as quantitative because it involves the collection of measurable data from 459 user reviews, where each review is assigned a sentiment score (positive, negative, or neutral). This scoring process transforms qualitative opinions into quantifiable categories for the presentation of results in numerical terms, such as the percentages of positive versus negative sentiments.

**Comment 3:** Line 196. "was to identify how users feel about apps."- elaborate specifically, what do you mean by how users "feel" about apps? feeling is an emotion, are exploring their emotions? perhaps there are better terminologies than the "feeling" of users.

**Response:** Thank you for your insightful comment. By "feel," we refer to users' perceptions and attitudes towards the apps, encompassing their satisfaction, usability experiences, and overall sentiments. We aim to explore both emotional responses and evaluative judgments that users express in their reviews. To enhance clarity, we revised the terminology in the manuscript to "user perceptions" instead of simply "feel."

**Comment 4:** Both figures 1 and 2 are blurry and unclear. I could not read the texts, even when downloaded. Please provide a better quality of the figures.

**Response:** Thank you for your comment. We have improved the quality of Figure 1 and redesigned Figure 2, which has now been divided into two separate figures (Figure 2 and Figure 3). Additionally, we included the percentage for each review category to enhance clarity and readability.

**Comment 5:** Results. Please provide the quotations that support the themes. Every theme must be backed with the quotation of the reviews. Please provide the demographic profile of reviewers if possible.

**Response:** Thank you for your valuable feedback. We want to clarify that each category in our results section is supported by one quotation from the data as an example, illustrating the themes identified. However, regarding the request for a demographic profile of reviewers, we have decided to focus on thematic analysis in this manuscript. We appreciate the suggestion and will consider incorporating demographic information in future research.

We have made all the suggested revisions and highlighted the changes in the manuscript for your convenience. Thank you once again for your insightful comments. We believe these revisions have significantly improved the quality of our manuscript and look forward to your feedback on the revised version.
